# Bipolarons rule the short-range terahertz conductivity in electrochemically doped P3HT†

**DOI:** 10.1039/d1mh01343b

**Published:** 2021-12-07

**Authors:** Demetra Tsokkou, Priscila Cavassin, Gonzague Rebetez, Natalie Banerji

**Affiliations:** Department of Chemistry, Biochemistry and Pharmaceutical Sciences (DCBP), University of Bern, Freiestrasse 3 3012 Bern Switzerland natalie.banerji@unibe.ch

## Abstract

Doping of organic semiconductor films enhances their conductivity for applications in organic electronics, thermoelectrics and bioelectronics. However, much remains to be learnt about the properties of the conductive charges in order to optimize the design of the materials. Electrochemical doping is not only the fundamental mechanism in organic electrochemical transistors (OECTs), used in biomedical sensors, but it also represents an ideal playground for fundamental studies. Benefits of investigating doping mechanisms *via* electrochemistry include controllable doping levels, reversibility and high achievable carrier densities. We introduced here a new technique, applying *in situ* terahertz (THz) spectroscopy directly to an electrochemically doped polymer in combination with spectro-electrochemistry and chronoamperometry. We evaluate the intrinsic short-range transport properties of the polymer (without the effects of long-range disorder, grain boundaries and contacts), while precisely tuning the doping level *via* the applied oxidation voltage. Analysis of the complex THz conductivity reveals both the mobility and density of the charges. We find that polarons and bipolarons need to co-exist in an optimal ratio to reach high THz conductivity (∼300 S cm^−1^) and mobility (∼7 cm^2^ V^−1^ s^−1^) of P3HT in aqueous KPF_6_ electrolyte. In this regime, charge mobility increases and a high fraction of injected charges (up to 25%) participates in the transport *via* mixed-valence hopping. We also show significantly higher conductivity in electrochemically doped P3HT with respect to co-processed molecularly doped films at a similar doping level, which suffer from low mobility. Efficient molecular doping should therefore aim for reduced disorder, high doping levels and backbones that favour bipolaron formation.

## Abstract 2

*In situ* terahertz spectroscopy on electrochemically doped P3HT reveals the impact of polarons and bipolarons on the short-range conductivity.

## Graphical abstract

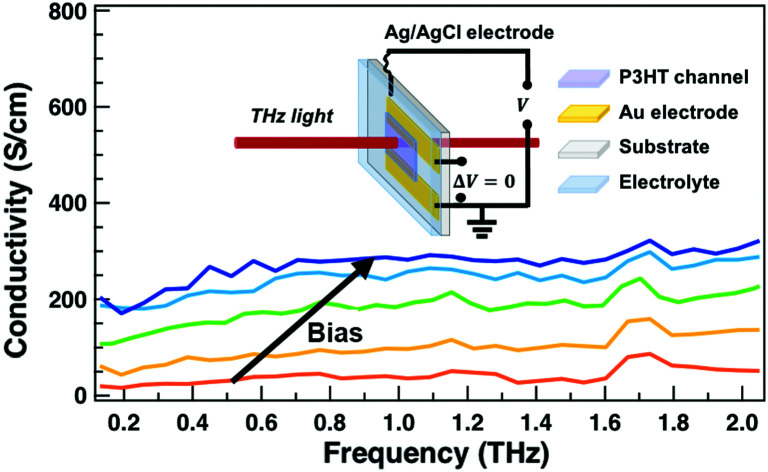

**New concepts**

We introduce a challenging new experimental technique, whereby we measure the complex terahertz (THz) conductivity in electrochemically doped polymers under varied applied bias. This allows to unravel unprecedented and quantitative understanding about the intrinsic few-nanometer conductivity as a function of injected charge density and the neutral:polaron:bipolaron ratio. We demonstrate that bipolarons play an essential role in maximizing the local conductivity and that they need to co-exist with polarons in an optimal ratio in order to reach up to 300 S cm^−1^. Above a threshold bipolaron density, the further increase in conductivity is not related to a rise in the charge mobility, but rather to an enhanced fraction of injected charges contributing to the transport by hopping between bipolaronic and polaronic sites.

## Introduction

Organic semiconductors such as conjugated polymers are emerging as a viable alternative to their widely commercialized inorganic counterparts, due to their excellent optical and electronic characteristics, combined with chemical/structural tunability, favorable mechanical properties and solution-processability.^1,2^ A drawback currently limiting the performance of organic materials is their low electronic conductivity. This can be increased by several orders of magnitude by adding extra positive or negative charge to the conjugated polymer backbone, doping the material either electrochemically or *via* use of molecular dopants.^3–6^ Doped conjugated polymers have shown great promise in electrochromic windows, optoelectronics, thermoelectrics and bioelectronics.^3,4^ Various approaches have been developed in molecular doping, such as co-mixing the polymer and dopant in solution before film deposition or sequentially adding the dopant *via* vapor or solution phase onto the polymer film.^4,7^ The molecular dopant plays a double role. First, it undergoes charge transfer with the conjugated polymer leading to the formation of conductive charges and second, the ionized dopant is needed to compensate the charges on the polymer backbone. Conjugated polymers exhibiting mixed ionic-electronic conduction are increasingly employed in devices based on electrochemical doping.^8^ Here, the doping level of the organic film immersed in an electrolyte solution is varied by electrochemical redox reactions at different applied voltages.^6,9^ The charges transferred to the polymer backbone from an electrode are distributed throughout the entire bulk of the film and are counterbalanced by ions that penetrate the film from the electrolyte.^6,9^

Mixed electronic-ionic conduction is intensively studied in the context of organic electrochemical transistors (OECTs), especially in aqueous environments. OECTs are used for the electric stimulation of cells, biosensing and in neuromorphic devices, and investigations on electrochemical doping have nowadays extended towards supercapacitors and batteries.^5,6,10,11^ PEDOT:PSS (poly(3,4-ethylenedioxythiophene):poly(styrenesulfonate)) is the prototypical material used for electrochemical devices operated in aqueous electrolytes, whereby the initial state is doped and becomes de-doped when a voltage is applied (depletion mode).^12,13^ In the past years, interest has risen to electrochemically dope initially un-doped polymer films, both p-type and n-type, since operation in accumulation mode requires less power consumption in OECTs.^14–19^ Here, we investigate electrochemical doping of poly(3-hexylthiophene) (P3HT), a well-characterized p-doped polymer and workhorse material for studying doping processes in conjugated polymers. We compare this to molecularly doped P3HT films. From a fundamental viewpoint, the benefits of studying doping mechanisms *via* electrochemistry include the controllable doping level, the reversibility of the doping/dedoping processes and the high charge carrier concentrations (exceeding 10^21^ cm^−3^) achievable at low operating voltages, which are much less accessible when molecular dopants are used.^20–22^ In electrochemically doped P3HT, a high macroscopic conductivity of >200 S cm^−1^ has been recently reported,^23^ which is close to the one obtained in molecularly doped highly oriented P3HT films, where the high polymer crystallinity allows for high conductivities.^24,25^ In the electrochemically doped polymer, polarons (singly charged carriers) and bipolarons (doubly charged carriers that form zero spin states) are known to form at different oxidation voltages; a clear picture of the role of bipolarons on charge transport and how they facilitate or inhibit high conductivities is missing.^23,26–28^ Our motivation is to link the nature and density of the charged species (polarons, bipolarons) to the conductivity and transport properties of doped P3HT. We focus on the less investigated high doping regime that is mainly accessible *via* electrochemical doping.

To this aim, we use both *in situ* optical and terahertz (THz) spectroscopy at various applied voltages. Recently, the need to develop new *in situ* measurements for understanding mixed ionic-electronic conduction in electrochemical devices, such as OECTs, has been realized. One of the first techniques that was reported is the electrochromic moving front experiment on planar electrolyte-polymer interfaces that yields the ion mobility in polymer films.^13^*In situ* electrochemical strain microscopy on OECT devices distinguishes between surface and volumetric doping by probing even sub-nanometer changes in the polymer film thickness.^29^ The role of polymer swelling from water and anions in mixed conductors containing hydrophilic glycolated side chains was accessed *via in situ* electrochemical quartz crystal microbalance monitoring,^15,16^ while structural changes during electrochemical reactions can be addressed by *in situ* X-ray scattering experiments,^30^ and *in situ* spectro-electrochemistry allows to determine the presence of different polymer redox species.^18,30,31^ We now add *in situ* THz spectroscopy to this palette, since this measures the short-range charge transport properties,^32,33^ which have been far less discussed than the long-range transport.^22–24,34^ This is important to consider because of the highly dispersive transport in conjugated polymers, which arises from large energetic disorder and weak intermolecular electronic interactions.^35^ In general, charge mobility is high along the polymer chains and between well-packed chain segments, but it is much lower over longer distances (due to less well-packed chains, traps and grain bounderies).^36^ Using contactless THz spectroscopy, we selectively probe the motion of mobile charge carriers within the short duration (∼1 ps) of the THz pulse. Thus, we are sensitive to fast transport events occurring over a few nanometers.

By applying for the first time *in situ* THz spectroscopy, spectro-electrochemistry and chronoamperometry to a P3HT film, we find that the co-existence of polarons and bipolarons in an optimal ratio allows a higher concentration of carriers to participate in the transport, which enhances the conductivity. Moreover, we compare the local transport properties between electrochemically doped and co-processed molecularly doped P3HT at a similar doping level and show significantly higher conductivity in the former case, which is related to higher charge mobility and delocalization.

## Results and discussion

**Fig. 1:**
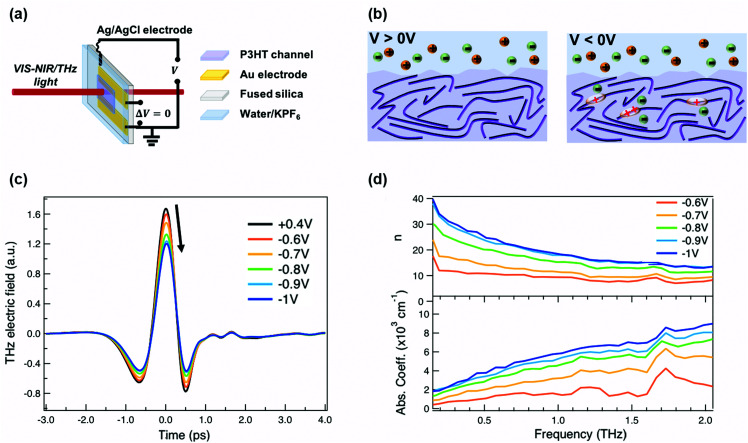
(a) Representation of the device structure used for the *in situ* absorbance and THz spectroscopy measurements. (b) Schematic of the electrolyte/polymer interface at different applied voltages *versus* Ag/AgCl. Anions (in this case PF_6_^−^) and cations (in this case K^+^) are shown in green and orange, respectively. At positive voltages, the polymer is in the undoped state. At negative voltages, polarons and/or bipolarons are formed on the P3HT polymer backbone depending on the applied voltage, together with the injection of anions into the polymer film that compensate the charge. (c) Transmitted time-domain THz pulse and (d) THz refractive index (top) as well as absorption coefficient (bottom) of electrochemically doped P3HT at different oxidation potentials.

For our measurements, a ≈200 nm P3HT film is spin-coated on parallel gold electrodes with a spacing of 5 mm (see Fig. 1a) and placed inside a spectro-electrochemical cell, where a thin layer (≈60 μm) of aqueous 0.1 M hexafluorophosphate (KPF_6_) electrolyte is added on top of the film together with an Ag/AgCl electrode.^6,20^ We have chosen this device architecture to allow both *in situ* absorbance and THz conductivity measurements to be carried out on the same sample. The gap is necessary to allow transmission in both spectral ranges, since even an optically transparent ITO electrode would block or severely distort the THz beam. Unlike for a true three-terminal OECT configuration, the two parallel gold contacts are short-circuited during the measurements in order to avoid lateral inhomogeneity of the doping within the P3HT channel. Since the primary goal of our study is to determine the conductivity of the P3HT film at different doping levels, averaging with our 3 mm diameter THz beam over a doping gradient (which occurs across the channel during OECT operation) would only complicate the analysis. We have chosen PF_6_^−^ anions to counterbalance the positive polarons formed on the P3HT backbone, because of the high doping levels achieved, the doping reversibility and the weak film swelling during doping.^14,15,18^ The doping state of the polymer is electrochemically controlled by modifying the applied voltage *via* the Ag/AgCl electrode. After cycling 4–5 times between 0.4 V and −1 V *versus* Ag/AgCl to stabilize the film morphology upon PF_6_^−^ injection/extraction, the device is held for 180 s at each applied voltage to allow uniform counterion distribution in the polymer film prior to the steady-state spectroscopic measurements. When a positive voltage is applied (*V* > 0 V), the polymer is in its undoped state (sketched on the left of Fig. 1b). Upon application of a negative voltage (*V* < 0 V), holes are injected into the bulk of the P3HT film from the gold electrodes along with penetration of PF_6_^−^ counterions from the electrolyte for charge compensation (right of Fig. 1b).^6,20,37^ As the amplitude of the applied voltage increases, it drives more holes and PF_6_^−^ counterions into the film, allowing controllable adjustment of the polymer doping level.

The time-domain THz pulses transmitted through the P3HT device at different applied voltages are shown in Fig. 1c. The transmitted THz electric field shows a continuous decrease in amplitude and a phase shift with increasing negative voltage *versus* Ag/AgCl. This is related to the formation of polarons and bipolarons on the P3HT backbone, which absorb and delay the THz radiation. Details about the THz experiment and the data analysis are given in the ESI.† We assume a similar P3HT film thickness in the aqueous electrolyte as in the dry film, since passive swelling of the hydrophobic polymer by water molecules is not expected.^16^ Moreover, upon voltage application, a total swelling of only 0.5–1.5% has been reported for electrochemically doped P3HT in aqueous electrolyte.^18^ As the large PF_6_^−^ anions are weakly hydrated, they carry only 1–2 water molecules into the film during electrochemical doping.^14,18^ The anions themselves intercalate between the P3HT chains in amorphous regions (low voltage) or crystalline regions (high voltage) and do not significantly increase the film volume.^20,38^ Since film swelling and water penetration at different electrochemical doping levels are weak, we use a constant film thickness and neglect the effect of water inside the film during the THz analysis.

In Fig. 1d, the frequency-domain THz refractive index (*n*, top) and absorption coefficient (*k*, bottom) of electrochemically doped P3HT are shown, as extracted from the Fourier Transform of the transmitted THz pulses. Only voltages between −0.6 and −1 V *versus* Ag/AgCl are displayed since no significant changes are induced on the THz waveform at less negative voltages. As a consequence of doping, a high and dispersive THz refractive index is found, which is larger than the one reported for undoped polymers (usually ≈1.5–1.7).^39^ Conventional inorganic semiconductors such as Si or GaAs are usually studied at significantly lower doping concentrations (10^16^–10^17^ cm^−3^), which are sufficient to increase the conductivity by several orders of magnitude.^40^ In this case, a constant THz refractive index is seen for the doped and undoped state.^41,42^ However, in heavily doped inorganic materials, where the doping concentration approaches that of doped organic materials (≥10^19^ cm^−3^), a similar strong increase of the THz refractive index is observed as we see here.^43^ Also, higher absorption coefficients are caused by the formation of charges on P3HT.

**Fig. 2:**
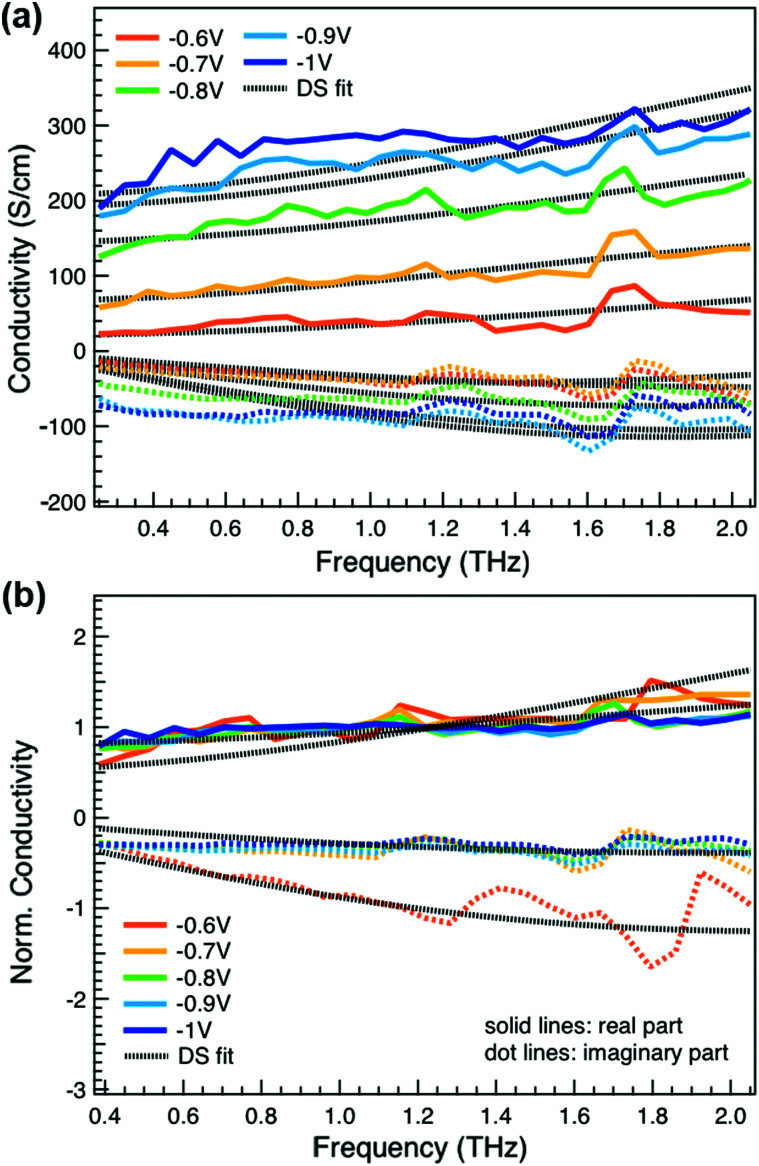
(a) Complex conductivity spectra of electrochemically doped P3HT at different oxidation potentials *versus* Ag/AgCl. The real and imaginary parts are shown with solid and dotted lines. (b) Normalized real (solid) and scaled imaginary (dashed) parts of the conductivity at different applied voltages. The fit results from the Drude–Smith model are shown with black dotted lines.

We obtain more information about the short-range charge carrier mobility, density and delocalization at different doping levels from the complex THz conductivity (calculated from *n* and *k*) shown in Fig. 2a, where the real and imaginary parts are depicted with solid and dashed lines, respectively. The slightly increasing real part of the conductivity with frequency and the negative imaginary part seen at all doping levels suggest that the mobile charge carriers are localized over the length scale of a few nanometers that we probe *via* the THz measurements. In conjugated polymers, this is caused by local disorder due to bending/twisting of the polymer backbone, chain entanglements and the finite chain length, which act as energetic barriers for charge transport and result in incoherent hopping events even over short distances.^44,45^ Similar THz behavior has been reported for molecularly doped P3HT,^32^ PEDOT:PSS,^33^ and for charge carriers in photoexcited polymer:fullerene blends.^44,46,47^ We find here that the real conductivity continues to increase up to −1 V *versus* Ag/AgCl, where the maximum is ≈300 S cm^−1^.

**Fig. 3:**
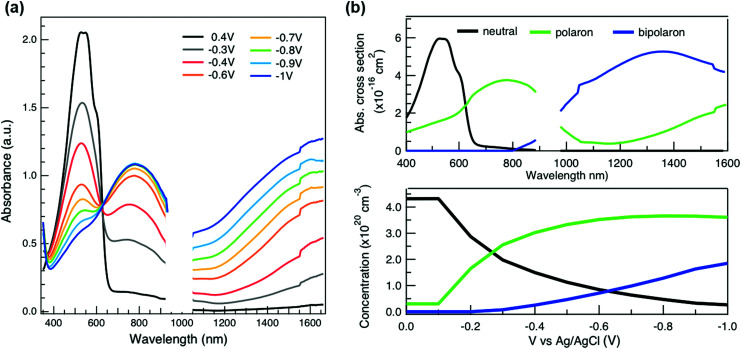
(a) *In situ* absorbance spectra of the spin-coated P3HT film at different applied voltages *versus* Ag/AgCl electrode in 0.1 M KPF_6_/water in steady state conditions. (b) Component spectra (top) and voltage-dependent concentrations (bottom) of the neutral, polaronic and bipolaronic species of electrochemically doped P3HT, as obtained from soft-modeling MCR-ALS analysis of the spectro-electrochemistry spectra.

We compare the real conductivity spectra normalized at 1 THz and the respective scaled imaginary part (divided by the same constant) in Fig. 3a, to evaluate changes in the shape of the complex conductivity spectra with applied voltage. The real conductivity in electrochemically doped P3HT does not exhibit any significant differences, but the scaled imaginary part has a higher amplitude at −0.6 V. This indicates that the charged species, which are mainly polarons at low oxidation voltages, are more localized than those that contribute in the conductivity at higher doping levels. To quantify this observation, we use the phenomenological Drude–Smith model, which describes the behaviour of localized charge carriers in disordered media.^48^ Fits of the conductivity spectra are shown in Fig. 2 and the fitting parameters are given in Table 1. In the Drude–Smith model, the complex conductivity is described by:
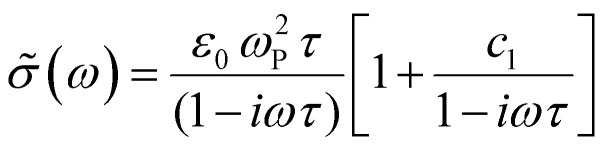
where *ω*_P_ is the plasma frequency, *ε*_0_ the free space permittivity, *τ* the scattering time and *c*_*1*_ the persistence of velocity or localization parameter. The latter is related to the probability of charge carriers to backscatter after a collision at boundaries. This parameter varies between 0 and −1, where 0 restores the Drude model and reveals the presence of free charges, while the lowest limit describes strongly localized charges. Factors that define the localization parameter in organic semiconductors include the charge delocalization, the finite polymer chain size and the energetic disorder.

**Table 1:** Fitting parameters from the Drude–Smith model-based analysis of the complex THz conductivity spectra in electrochemically doped P3HT and co-processed P3HT:17% mol F_4_TCNQ films

*V versus* Ag/AgCl (V)	*ω*_P_ (THz)	*N*_DS_ (× 10^19^ cm^−3^)	*τ* (fs)	*c*_1_
−0.6	303	4.9	30	−0.91
−0.7	372	7.4	30	−0.78
−0.8	488	12.7	30	−0.77
−0.9	572	17.5	30	−0.78
−1	590	18.6	30	−0.78
P3HT:17% mol F4TCNQ	426	9.7	13	−0.96

The scattering time is related to very local band-like transport in polymer chain segments or aggregates.^46^ Given the similar shape of the real conductivity spectra at all applied voltages, we have fixed this parameter. The value of 30 fs that we find is usual for conjugated polymers,^49,50^ where charge delocalization is rather limited (Table 1). As discussed above, the energetic barriers present in conjugated polymers result in incoherent hopping events even for motion over short distances. Therefore, a negative localization parameter is observed at all voltages. We note a stronger localization at −0.6 V than at higher oxidation voltages. By assuming an effective mass of m* = 1.7 *m*_e_ as used previously for P3HT,^51^ we calculate the effective THz mobility 
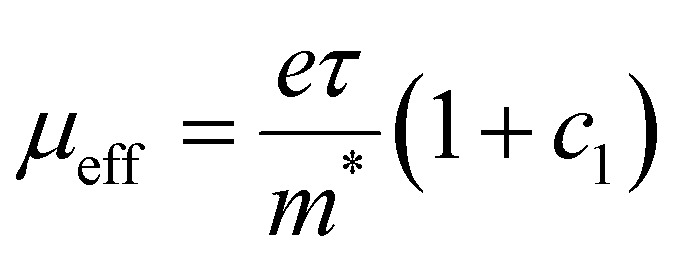
 over the nanometer length scale.^47^ A higher mobility of *μ*_eff_ ∼6.8–7.1 cm^2^ V^−1^ s^−1^ is obtained at higher applied voltages (−0.7 V to −1.0 V *versus* Ag/AgCl), caused by the decrease of the localization parameter from *c*_1_ = −0.91 at −0.6 V to *c*_1_ = −0.78 at −1 V. Moreover, the continuous increase of the conductivity upon doping is explained by the increase of the mobile charge carrier density *N*_DS_, which is related to the squared plasma frequency extracted by the Drude–Smith model 
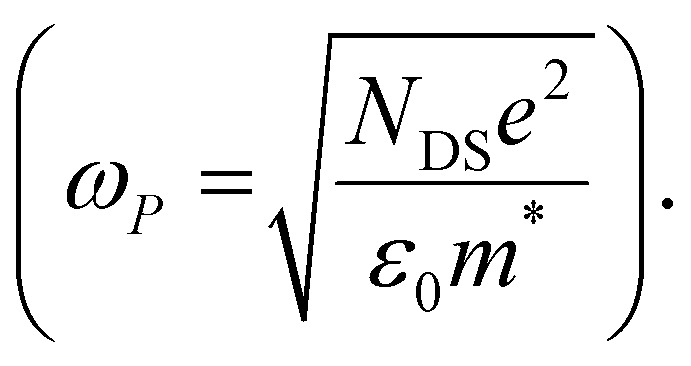


To relate the evolution of the THz conductivity to the nature and density of the conductive species (polarons, bipolarons) formed upon oxidation of P3HT at different doping levels, we carried out *in situ* spectro-electrochemistry in the visible to near-infrared (VIS-NIR) range on the same P3HT device. The spectra at different applied voltages are shown in Fig. 3a. At positive voltage (0.4 V), the neutral P3HT absorption (400–600 nm) dominates, while the weak signature in the NIR region due to polarons shows that the polymer film remains weakly doped after the initial cycling. At negative voltages, the P3HT film switches from its neutral to its oxidized form, as revealed by the depletion of the neutral P3HT band and the progressive rise of the NIR absorbance bands due to polarons and bipolarons. To disentangle the spectra associated to the different species that co-exist at each doping level, we analyze the absorbance spectra using soft-modeling multivariate curve resolution (MCR-ALS) analysis.^52,53^ Three components with different spectral features are unraveled (top of Fig. 3b), which can be clearly assigned to the neutral, polaronic and bipolaronic species based on existing literature: The spectrum of neutral P3HT (shown in black) has a vibronic peak at 610 nm that is characteristic of crystalline P3HT regions.^54,55^ At negative voltages, the neutral band decreases in amplitude due to the doping process and the vibronic structure vanishes (Fig. 3a). This occurs because the crystalline regions are doped at lower oxidation voltages compared to the amorphous ones, so that the unstructured signature of amorphous neutral P3HT persists at more negative voltages.^20,21,56,57^ The polaron component (shown in green in Fig. 3b) peaks at ∼780 nm (P_2_ band), and has a second transition (P_1_ band) rising above 1200 nm.^23,57,58^ The bipolarons (blue spectrum) absorb at wavelengths longer than 900 nm with a peak at 1350 nm.^23,58,59^ Using the total injected charge at each voltage (from integrated chronoamperometry measurements, see ESI†), the absorption cross section of the spectral components is determined, allowing to obtain the concentrations of the three species as a function of voltage (bottom of Fig. 3b). Between −0.1 V and −0.4 V, mainly the decay of neutral P3HT and rise of polarons occurs. At higher oxidation voltages (−0.4 V to −1 V), bipolarons are gradually formed, while the density of neutral species continues to decrease and the one of the polarons saturates. These findings agree with previous results on electrochemically doped P3HT films.^1,2^

**Fig. 4:**
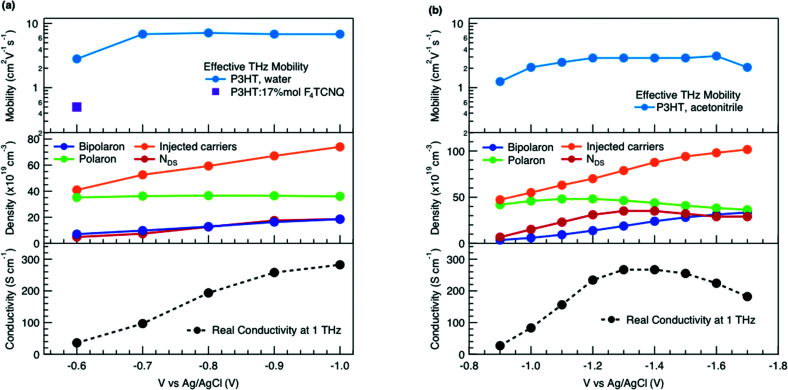
Parameters extracted from the THz and spectro-electrochemistry data as a function of applied voltage *versus* Ag/AgCl for electrochemically doped P3HT in (a) KPF_6_/water and (b) TBAPF_6_/acetonitrile electrolyte. In the top panel, the THz effective mobility is shown, the middle panel compares the density of the charged species (as extracted *via* MCR-ALS analysis) to the total injected charges and the concentration of conductive charges (*N*_DS_, from the squared plasma frequency, Drude–Smith model), while the bottom panel depicts the real conductivity at 1 THz. In (a) the THz effective mobility of molecularly doped P3HT is also shown.

As discussed above, there is a continuous increase of the real THz conductivity with increasing oxidation voltage (Fig. 4a). The conductivity depends on both the number of mobile charge carriers as well as their mobility. An increase of the effective THz mobility occurs only between −0.6 V and −0.7 V, as the c_1_ localization parameter becomes less negative. At more negative voltages, the rise in conductivity is caused solely by the increase of charge density in the P3HT film and increases with the injected charge density obtained from the integrated current transients (Fig. 4a). We note that not all injected charges contribute to the THz conductivity, since not all carriers within the polaronic and bipolaronic density of states (DOS) are sufficiently mobile. Therefore, the density of mobile charges derived from the squared plasma frequency (*N*_DS_) is about 4–8 times lower than the total injected charge density. By considering the polaron and bipolaron concentrations from spectro-electrochemistry, it becomes clear that the charge injection mainly leads to the formation of bipolarons in the considered voltage range (Fig. 4a), while the polaron concentration increases by less than 10% and saturates at −0.7 V. Indeed, *N*_DS_ closely follows the bipolaron density, showing that each bipolaron (corresponding to the injection of two charges) adds one charge to the conductivity. The mobile charges (*N*_DS_) also represent a larger fraction of the total charge density as more bipolarons are formed (increase from 12% to 25%). This could either be because the bipolarons themselves are highly conductive, or because the coexistence of polarons and bipolarons is beneficial. The fact that the mobility stays constant after −0.7 V, although the bipolaron density continues to rise, suggests that the bipolarons are not more mobile than the polarons. Rather, the presence of bipolarons above a threshold concentration of about 20% increases the mobility of the conductive charges (less negative localization parameter) and allows more charges to participate in the transport.

Several factors can contribute to this effect at high oxidation voltages. First, the bipolarons induce structural changes in polythiophene films (as has been observed *in situ* for electrochemical PEDOT:PSS reduction),^30^ which might lead to better packing and short-range conductivity compared to −0.6 V, where a non-negligible fraction of neutral sites (∼20%) add conformation barriers and localize the charges. The infiltration of counterions into the crystalline regions of P3HT at high doping levels can cause a similar morphological increase of the mobility, as has been reported in electrochemically doped polymers gated with an ionic liquid.^20^ Second, at high charge density, a regime can be reached where the carriers (and especially the bipolarons) shield the electrostatic attraction to the negative counterions, whose Coulomb potentials start to overlap, leading to reduced trapping and increase of the conductivity (but likely we are already in this regime at −0.6 V).^60–62^ Third and most importantly, the coexistence of polarons and bipolarons allows mixed valence conduction, whereby the hole hops from bipolaronic to polaronic redox sites during transport.^23,63,64^ This is more efficient than hopping between polaronic and neutral sites, since important conformational changes occur between the twisted neutral form and more planar polaronic form of P3HT, whereas the configuration of polarons and bipolarons is more similar. Thus, a higher density of charge carriers participates in the transport when both polarons and bipolarons are present. According to this model, the conductivity should decrease when the concentration of bipolarons starts exceeding the one of polarons. Indeed, macroscopic conductivity measurements carried out *in situ* on electrochemical devices of P3HT and other polymer films typically show a plateau or decrease of the conductivity at high applied voltages.^65^

In our THz data, both the squared plasma frequency and the real conductivity seem to saturate at the highest oxidation voltage (when 33% of the carriers are bipolarons, Fig. 4a). To verify this, we have repeated the spectro-electrochemistry and *in situ* THz measurements using 0.1 M TBAPF_6_ in acetonitrile as the electrolyte, since this allows to achieve higher doping levels. Now the onset of bipolaron formation is shifted to −0.9 V *versus* Ag/AgCl, but a higher bipolaron density than in water of almost 50% can be reached at −1.7 V, together with a complete depletion of the neutral species and a decay of the polarons due to conversion to bipolarons (Fig. S3, ESI†). As in the aqueous electrolyte, an increase in carrier mobility due to enhanced delocalization (less negative *c*_1_) is seen only at low oxidation voltages, then the mobility stays constant (Fig. 4b, Fig. S4 and Table S1, ESI†). The short-range THz conductivity keeps increasing until −1.3 V (where the total bipolaron concentration is around 30%, comparable to the maximum concentration in KPF_6_/water). This follows the trend in total injected charge, which leads to the formation of both polarons and bipolarons and a steep increase in the mobile carrier density *N*_DS_. The short-range conductivity at −1.3 V (270 S cm^−1^) is slightly higher than the macroscopic conductivity reported when P3HT is electrochemically doped in TBAPF_6_/acetonitrile electrolyte,^23^ as expected due to dispersive transport in conjugated polymers.^66^ Both the conductivity and *N*_DS_ then decrease at voltages beyond −1.3 V, despite further injection of charges leading to an increase of the bipolaron concentration. The trend is accompanied by a decrease of polaron density. This evidences that the short-range conductivity in electrochemically doped P3HT is dominated by hopping between polaronic and bipolaronic sites, so that the fraction of mobile carriers participating to transport decreases as bipolarons are surrounded by other bipolarons rather than by polarons. At such high charge densities, electrostatic repulsions between carriers also limit the transport.^67^

**Fig. 5:**
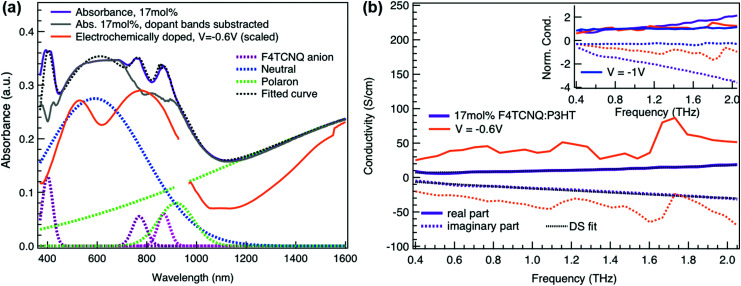
(a) Absorbance spectrum of molecularly doped P3HT:17 mol%F_4_TCNQ film and of the electrochemical device at −0.6 V *versus* Ag/AgCl (scaled). The molecularly doped spectrum was decomposed into Gaussian components representing the neutral, polaron and bipolaron species as well as the ionized dopant. The polymer and dopant were co-processed in solution before film deposition. (b) Complex conductivity spectra of the molecularly doped film compared to the electrochemical sample. The real and imaginary parts are shown with solid and dotted lines. The inset shows the normalized real part and scaled imaginary part of the conductivity.

Finally, to compare the properties of electrochemically and molecularly doped P3HT, we study a doped P3HT:17 mol% F_4_TCNQ film, where the polymer and F_4_TCNQ dopant are co-processed in solution. This dopant concentration corresponds to the maximum reported doping level.^68^ At higher dopant concentrations, neutral dopant aggregates are formed already in solution which deteriorate the film morphology and prevent efficient doping.^68^ The absorption spectrum of the molecularly doped film is shown in Fig. 5a. In addition to the bands of neutral and oxidized P3HT, peaks at ∼400 nm, ∼760 nm and ∼860 nm are seen, which arise from the ionized F_4_TCNQ molecules.^68,69^ Based on a Gaussian decomposition of the spectrum (Fig. 5a) and the known absorption cross section of the 760 nm band (7.65 × 10^−17^ cm^2^),^69^ we estimate an ionized dopant concentration of 3.4 × 10^20^ cm^−3^, corresponding to the total charge density on the P3HT backbone. The (bi-)Gaussian signatures of the neutral, polaron and bipolaron bands resemble the ones seen in electrochemically doped P3HT. The total charge density and the ratio between the three species are comparable to the electrochemically doped sample in aqueous electrolyte when a voltage of *V* = −0.6 V *versus* Ag/AgCl is applied. Despite the comparable doping level, the THz conductivity of molecularly doped P3HT is lower than the one of the electrochemically doped polymer at −0.6 V (∼10 S cm^−1^*vs.* ∼36 S cm^−1^ at 1 THz) and the conductivity of 300 S cm^−1^ measured at −1 V *versus* Ag/AgCl is inaccessible using the co-processed molecular doping protocol (Fig. 5b).

The Drude Smith analysis reveals that the charge carrier localization differs between the two doping approches. A localization parameter close to −1 (*c*_1_ = −0.96) is extracted in the molecularly doped film, while in electrochemically doped P3HT, *c*_1_ = −0.91 (at *V* = −0.6 V *versus* Ag/AgCl) and *c*_1_ = −0.78 at higher oxidation potentials (Table 1). The higher charge localization for molecular doping agrees with the enhanced scaled negative imaginary conductivity (Fig. 5b inset). This in combination with the shorter scattering time (∼13 fs) results in charge carriers with significantly lower effective mobility (*μ*_eff_ ∼0.5 cm^2^ V^−1^ s^−1^ compared to *μ*_eff_ ∼2.8 cm^2^ V^−1^ s^−1^ in the electrochemical device at −0.6 V). Hence, since similar doping levels are compared, the lower charge carrier mobility is the factor limiting the THz conductivity in the molecularly doped P3HT film. This is related to the higher static energetic disorder in molecularly doped P3HT, arising from aggregation of polarons and ionized dopants already in solution that results in a poorly connected solid-state nanostructure.^7,34^ In contrast to molecularly doped P3HT, the electrochemically doped polymer film exhibits weaker morphological disruptions by ion injection, leading to lower energetic disorder and higher charge delocalization. Similar conclusions are obtained from studies on long-range transport in molecularly sequentially doped polymer films, where an improved microstructural order allows higher mobilities.^54,70,71^ Moreover, Coulomb trapping of the charges by the ionized dopant might be enhanced compared to trapping by the electrochemical counterions,^72^ but this is expected to play a lesser role in the high doping regime investigated here.^34^

## Conclusion

In conclusion, we present here the first *in situ* THz conductivity study of electrochemically doped P3HT. This contactless technique allows to evaluate the intrinsic short-range transport properties of the polymer, while precisely controlling the doping level *via* the applied oxidation voltage. We combine this with VIS-NIR spectro-electrochemistry and chronoamperometry on the same device, so that we are able to relate the concentration of polarons and bipolarons at different voltages to the local nanometre-scale conductivity. We find that high THz conductivity (∼300 S cm^−1^) and mobility (∼7 cm^2^ V^−1^ s^−1^) is achieved in aqueous KPF_6_ electrolyte when polarons and bipolarons coexist. The charge mobility and delocalization initially increase up to a threshold concentration where bipolarons constitute ∼20% of the charged species, then those parameters stay constant. However, the conductivity continues to rise due to an increase in the density of conductive carriers. Not only are more carriers injected, but the fraction of the total charges participating to the transport rises from 12% to 25%. We attribute this mainly to effcicient hopping between polaronic and bipolaronic sites (mixed valence conduction). When the bipolaron density exceeds ∼30–35% (possible in TBAPF_6_/acetonitrile electrolyte), a drop in conductivity is observed, since hopping between solely bipolaronic sites is less favorable and hole carrier repulsion occurs. We note that we report here on average densities and conductivities and will discuss in a future study how they differ in crystalline and amorphous phases of P3HT.

While the high conductivity of electrochemically doped P3HT can to some extent be transferred to the solid state,^23^ it is important to establish design rules for stable molecularly doped conjugated polymer films with high conductivity. We show here that molecularly doped P3HT that is co-processed with F_4_TCNQ in solution suffers from a reduced THz mobility and lower charge delocalization compared to the electrochemically doped film at similar doping level. In addition to this detrimental effect of disorder on the conductivity, the molecular doping does not allow high enough levels to optimize the polaron-to-bipolaron ratio. Molecular doping strategies should therefore aim for reduced disorder (as is already done by sequential or ion-exchange doping),^54,70–72^ a high dopant solubility (to achieve high doping levels) and backbones that favour bipolaron formation, *i.e.* where the energetics and morphology allow for the charge-lattice coupling to compensate the repulsion between the two charges.

## Conflicts of interest

The authors declare no competing financial interest.

## Supplementary Material

MH-009-D1MH01343B-s001
