# Correction: Bipolarons rule the short-range terahertz conductivity in electrochemically doped P3HT

**DOI:** 10.1039/d1mh90069b

**Published:** 2022-01-10

**Authors:** Demetra Tsokkou, Priscila Cavassin, Gonzague Rebetez, Natalie Banerji

**Affiliations:** Department of Chemistry, Biochemistry and Pharmaceutical Sciences (DCBP), University of Bern, Freiestrasse 3 3012 Bern Switzerland natalie.banerji@unibe.ch

## Abstract

Correction for ‘Bipolarons rule the short-range terahertz conductivity in electrochemically doped P3HT’ by Demetra Tsokkou *et al.*, *Mater. Horiz.*, 2022, DOI: 10.1039/d1mh01343b.

The authors regret that the wrong version of Fig. 5 was presented in the published manuscript. The correct Fig. 5 is shown below.

**Fig. 5:**
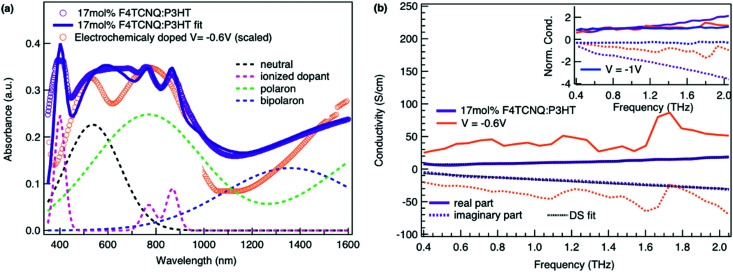
(a) Absorbance spectrum of molecularly doped P3HT:17 mol%F_4_TCNQ film and of the electrochemical device at −0.6 V *versus* Ag/AgCl (scaled). The molecularly doped spectrum was decomposed into Gaussian components representing the neutral, polaron and bipolaron species as well as the ionized dopant. The polymer and dopant were co-processed in solution before film deposition. (b) Complex conductivity spectra of the molecularly doped film compared to the electrochemical sample. The real and imaginary parts are shown with solid and dotted lines. The inset shows the normalized real part and scaled imaginary part of the conductivity.

The Royal Society of Chemistry apologises for these errors and any consequent inconvenience to authors and readers.

## Supplementary Material

