# Supplementary material for: Bipolarons rule the short-range terahertz conductivity in electrochemically doped P3HT
Source: Mater Horiz. 2021 Dec 7;9(1):482–91. doi: 10.1039/d1mh01343b (PMC8725991; doi:10.1039/d1mh01343b)
Supplement: MH-009-D1MH01343B-s001 [file MH-009-D1MH01343B-s001.pdf]

Electronic Supplementary Material (ESI) for Materials Horizons.
This journal is © The Royal Society of Chemistry 2021

Supporting Information

Bipolarons rule the short-range terahertz conductivity in electrochemically doped P3HT

Demetra Tsokkou[1], Priscila Cavassin[1], Gonzague Rebetez[1] and Natalie Banerji[1*]

[1]Department of Chemistry, Biochemistry and Pharmacy (DCBP), University of Bern, Freiestrasse 3, 3012 Bern, Switzerland.

S1. Experimental Methods

1. Sample preparation and characterization

P3HT Device Preparation: Chromium/Gold contacts were evaporated on quartz substrates previously cleaned in ultrasonic baths of Hellmanex (1% vol), bi-distilled water, acetone and isopropyl alcohol. The patterned substrates were submitted to a UV-O_3 treatment for 20 minutes at room temperature. A 40 mg/ml solution of P3HT (Ossila) in dichlorobenzene was spin-cast on the substrates at 700 rpm for 90 s. To define a 30 mm^2 channel between the two gold electrodes, precision tip cotton swabs were used. The sample was placed in a home-built spectro-electrochemical cell (made of PMMA for aqueous electrolyte or PCTFE for acetonitrile) together with an Ag/AgCl electrode and the electrolyte solution (0.1 mol L^{-1} KPF_6 in bi-distilled water or 0.1 mol L^{-1} $TBAPF_6$ in acetonitrile).

Molecularly doped P3HT film preparation: The P3HT and F_4TCNQ where dissolved and stirred separately in chlorobenzene for 2h at 60°C before mixing them together for 2h at 70°C. The polymer concentration in solution was chosen to be 10 mg/ml and the appropriate amount of F_4TCNQ was added to obtained 17 mol% relative to P3HT. For the purposes of the THz measurements that need thick samples (due to low conductivity), drop-casting was used and the co-processed solution was deposited into a Teflon mold that was left overnight to dry.

Film thicknesses were measured in a Bruker Contour GT-K optical profiler using white light interferometry and Vision 64 software for image acquisition.

2. *In-situ* spectroelectrochemical absorbance measurements in electrochemically doped P3HT

Our home-built spectroelectrochemical setup consists of a halogen light source (HL-2000, Ocean Insight), a Flame UV-Vis and a Flame NIR spectrometer (Ocean Insight) triggered by a digital delay/pulse generator (DG535, Stanford Research Systems), and a customized Labview code. The bias between the Ag/AgCl electrode and the gold electrodes was applied and measured with a data acquisition card (NI USB-6008, time

resolution of 7 ms). The transient response current was converted to voltage using a low-noise current preamplifier (SR570, Stanford Research Systems) and measured with the data acquisition card. Because the main focus of this work is about the effect of doping level on transport rather than about OECT performance, both gold contacts were shortcut for all measurements in order to ensure uniform doping of the channel. As a result, the voltage was always applied between the external Ag/AgCl electrode and symmetrically to both gold contacts. To increase reproducibility, the films were cycled for 5 times before all the experiments. One cycle consists of the application of a -1.0 V gate bias to dope the film for 30 s and a +0.4 V gate bias to de-dope the film for 30 s. The steady-state absorption spectra for different doping levels were collected by applying subsequent increasing steps of voltage, ranging from +0.2 V to -1.0 V. Two sets of measurements were collected. For the first, each voltage step was applied for 30 s, while for the second 120 s. Both show very similar results, which is an indication that steady-state conditions were reached.

The absorption spectrum of the co-processed P3HT:F_4TCNQ film was measured with a Lambda 950 UV-Vis-IR spectrophotometer (Perkin Elmer) with the use of an integrating sphere.

3. **Time-domain THz spectroscopy**

We use time-domain THz transmission spectroscopy to measure the intrinsic conductivity of doped polymers in the THz region. For the THz pulse generation, ultrashort light pulses are generated by a Ti:Sapphire amplifier system (Astrella, Coherent) that have a time duration of ~35 fs, 800 nm center wavelength, repetition rate of 1 kHz and energy of ~ 6 mJ/pulse. Part of the fundamental light is focused on a non-centrosymmetric ZnTe generation crystal via second order non-linear optical rectification. The generated THz pulses with pulse duration of about 1 ps are collimated and focused on the sample position via off-axis parabolic gold mirrors. The detection of the transmitted THz pulses is based on free space electro-optic sampling. This is composed of a quarter wave plate, a Wollaston prism and a balanced photodiode. The THz pulses transmitted via the sample are refocused on a second ZnTe crystals, where they spatially and temporary overlap with part of the fundamental pulses, which are named gate beam. During the propagation of the THz beam through the non-linear

crystal, it induces a change in the refractive index, also a second order non-linear optical effect, and the material becomes birefringent (Pockel effect). This rotates the polarization of the gate pulses that co-propagate with the THz beam through the crystal. The induced ellipticity in the optical pulses is probed by separating the two vertical polarization components with the quarter waveplate and the Wollaston prism, and their difference is measured in the balanced photodetector. The change in polarization is proportional to the amplitude of the THz electric field. By changing the time delay between gate and THz pulses via a motorized micrometer translation stage, the THz waveform is probed. This phase sensitive detection method allows to record the THz waveform and not just its amplitude, thus enabling the extraction of the complex optical properties. We apply a recent development of this detection scheme, where the polarization of the gate field is biased, allowing more than one order of magnitude increased sensitivity compared with the conventional electro-optic sampling detection scheme.[1] The bandwidth of the probed THz pulses is between 0.1 and 2.5 THz, which is limited due to phase mismatching in the non-linear crystals. The THz path is continuously purged with dry nitrogen to eliminate moisture that strongly absorbed by the THz radiation. For the electrochemically and molecularly doped materials studied here, we measure the ground state complex conductivity, therefore no photoexcitation is used.

---

[1]Krauspe, P.; Banerji, N.; Réhault, J., Effective detection of weak terahertz pulses in electro-optic sampling at kilohertz repetition rate. J. Opt. Soc. Am. B 2020, 37, 127

S2. Analysis of time domain THz measurements

To obtain the complex optical constants and therefore also the complex conductivity, two different THz measurements are necessary.

Molecularly doped P3HT: For the case of films, as in molecularly doped P3HT, we record the reference THz waveform in the first measurement, which is obtained from the THz pulse propagation through the quartz substrate. In the second measurement named the signal, the THz waveform transmits through the doped film and the substrate. Because of the polarons present in the doped film, the THz pulse is partly absorbed and delayed by the sample and these changes of the THz waveform are used for the calculation of the complex optical constants. As a first step, the frequency dependence of both the reference and signal THz amplitude and phase is calculated via fast Fourier transform. The following analysis then enables the direct extraction of both the real and imaginary part of the refractive index and the complex conductivity spectrum.[2] From the frequency-dependent spectra, the experimental complex transmission function $T_{exp}(\omega)$ is calculated, which is defined as the ratio between the THz waveforms propagated through the doped film $E_{sig}(\omega)$ (signal waveform) and substrate $E_{ref}(\omega)$ (reference waveform). Moreover, the complex transmission function $T_{mod}(\omega)$ is modelled by including the Fresnel transmission and reflection coefficients, the propagation factors through the different layers and the multiple reflections of THz pulses between the front and back surfaces of the film.[2] We consider a film layer of thickness *d* that is placed between the air and substrate. The complex transmission spectrum is given by the following equation:

$$T_{mod}(\omega) = \frac{2\bar{n}_1(1+\bar{n}_2)}{(1+\bar{n}_2)(\bar{n}_1+\bar{n}_2)}\, exp\left[-i(\bar{n}_1 - 1)\frac{\omega d}{c}\right] FP(\omega) \qquad (1)$$

where $\bar{n}_i$ is the complex refractive indices of layer *i* ($\bar{n}_i = n_i + k_i$) , 1 is the film and 2 the substrate layer, *d* the film thickness, ω and *c* are the angular frequency and speed of light in a vacuum and $FP(\omega)$ is the frequency dependent contribution of Fabry-Perot reflections in the thin film.[2]

---

[2]Brauer, J. C.; Thorsmolle, V. K.; Moser, J. E., Terahertz time-domain spectroscopy study of the conductivity of hole-transporting materials. *Chimia* **2009,** *63* (4), 189-192
Duvillaret, L.; Garet, F.; Coutaz, J., A reliable method for extraction of material parameters in terahertz time-domain spectroscopy. *IEEE J Sel Top Quantum Electron* **1996,** *2* (3), 739-746

Electrochemically doped P3HT: A different expression is used for electrochemical transistors because of the additional front quartz window and electrolyte layers through which the THz pulse propagates (Scheme S1). The modelled transmission coefficient is modified accordingly to include the additional layers.

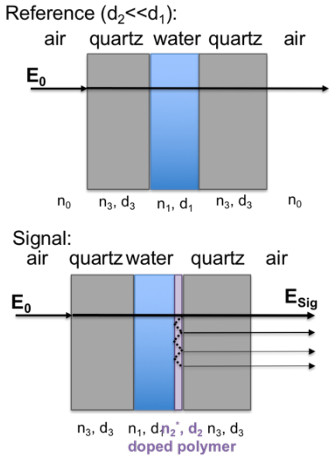

Scheme S1. Pathway of the reference (top) and signal (bottom) THz beam in the case of an electrochemical transistor device used for the THz measurements.

When an OECT device is used in THz spectroscopy, the spectral component of the THz electric field in the reference measurement is written as:

$$E_{Ref}(\omega) = T_{03}\, e^{i\omega d_3\, \bar{n}_3 d_3/c}\, T_{31}\, e^{i\omega d_1 \bar{n}_1 d_1/c}\, T_{13}\, e^{i\omega d_3 \bar{n}_3 d_3/c}\, T_{30}\, E(\omega)$$

where the electrolyte layer is indexed with 1, the doped polymer film with 2* and the quartz window is labeled with index 3. T_{ij} is the transmission coefficient between the layer *i* and *j* and is related to the complex refractive index of the layers ($T_{ij} = \frac{2\bar{n}_i}{\bar{n}_i+\bar{n}_j}$). Accordingly, the spectral component of the THz electric field in the signal measurement is given by:

$$E_{Sig}(\omega) = T_{03} e^{\frac{i\omega d_3 \bar{n}_3}{c}} T_{31} e^{\frac{i\omega d_1 \bar{n}_1}{c}}\, T_{12^*}\;\, e^{i\omega d_2 \bar{n}_2^*/c}\, FP(\bar{n}_1,\, \bar{n}_2^*,\, \bar{n}_3)\, T_{2^*3}\, e^{\frac{i\omega d_3 \bar{n}_3}{c}} T_{03} E(\omega)$$

The complex transmission coefficient is then given by,

$$T_{mod}(\omega) \;=\; \frac{E_{sig}}{E_{ref}} = \;\frac{2\bar{n}_2^*(\bar{n}_3+\bar{n}_1)}{(\bar{n}_2^*+\bar{n}_1)(\bar{n}_2^*+\bar{n}_3)}\;\, e^{i\omega d_2 \bar{n}_2 * \frac{c}{d}} * FP(\omega)$$

where d is the film thickness and $FP(\omega)$ is given by,

$$FP(\omega) = \frac{1}{1 - \frac{\bar{n}_2^* - \bar{n}_3}{\bar{n}_2^* + \bar{n}_3} * \frac{\bar{n}_2^* - \bar{n}_1}{\bar{n}_2^* + \bar{n}_1} e^{-2in_2^*\omega d/c}}$$

By performing a fit of the modelled $T_{mod}(\omega)$ to the experimental one, the real and imaginary refractive index ($\bar{n}(\omega) = n(\omega) + ik(\omega)$) spectra are derived. Then, the power absorption coefficient $\alpha(\omega)$ is calculated directly from the imaginary refractive index ($k(\omega) = \alpha(\omega)c/2\omega$). Lastly, the real σ_1 and imaginary σ_2 parts of conductivity are calculated via the complex refractive index and expressed as $\sigma_1 = 2\omega\varepsilon_0 nk$ and $\sigma_2 = \omega\varepsilon_0(k^2 - n^2)$, respectively, with ε_0 the permittivity of free space. For our measurements, quartz was used as substrate and window, with a THz refractive index $n(\omega) = 2.0$ nearly constant in the probed frequency range and a negligible absorption.

S3. Calculation of the refractive index of thew electrolyte in the THz region

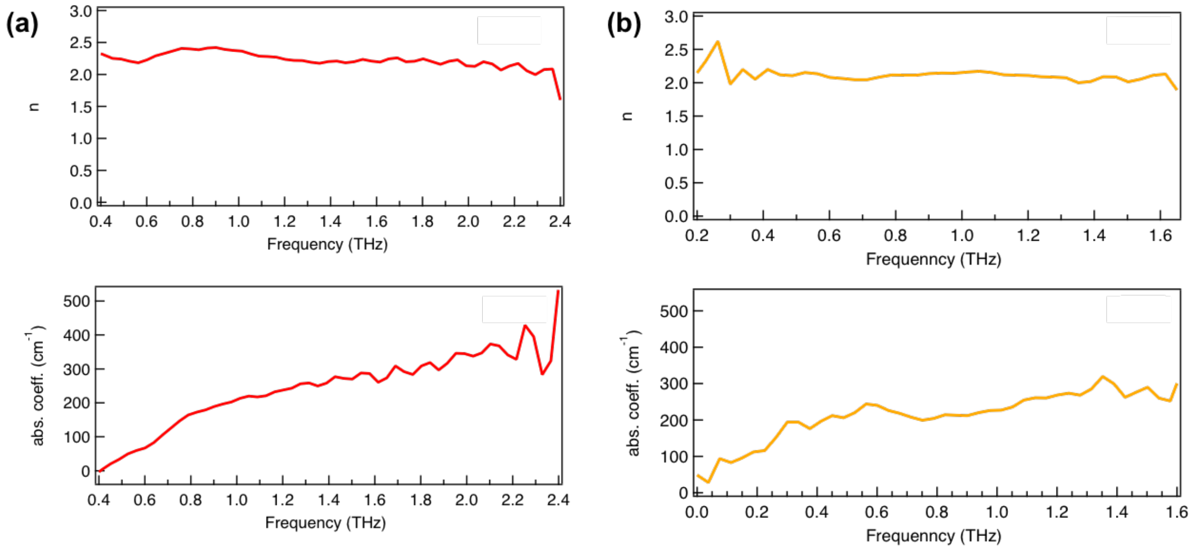

Figure S1. THz refractive index (n, top) and absorption coefficient (bottom) spectra of thin water/ KPF_6 layers. Layers of different thickness were used. Figures on the left side show the results of a 125 micron layer and on the right side the results for a 250 micron thick layer.

To be able to calculate the complex conductivity of electrochemically doped films, the refractive index of the electrolyte layer is necessary. Therefore, we performed measurements on an electrolyte layer placed between two quartz windows. In Figure S1, the spectra of the refractive index and of the absorption coefficient of the water/KPF_6 layer are shown. Measurements were repeated for two different thicknesses (d_1 = 125 microns, left part of Figure S1 and d_2 = 250 microns, right part of Figure S1) and similar results are found. The refractive index is between 2.2-2.3 and is found to be almost constant in this spectral window. A similar value was obtained for acetonitrile.

S4. Multivariate Curve Resolution (MCR) analysis

The data collected by spectro-electrochemistry measurements were analyzed with MCR analysis. The open-source software library *pyMCR* written in Python was used and the details of the method have been previously described elsewhere.[3] In short, the goal of the MCR is to decompose a matrix of data D in the product of two other matrixes, C and S, plus an E matrix that expresses the error. C stands for the species concentration and S for the species spectral shape. In this work, the MCR was used to decompose the unprocessed data into the spectral signatures and the concentrations of neutral, polaron and bipolaron species.

[3] H. Charles Jr. Camp, pyMCR: A Python Library for Multivariate Curve Resolution Analysis with Alternating Regression (MCR-AR), *J. Res. Natl. Inst. Stand. Technol.*, ***2019**, 124*, 124028

S5. Determination of absorption cross section

The concentrations obtained by the MCR analysis do not correspond to real concentrations unless the absorption cross sections of the species are known. The absorption cross sections of the neutral, polaron and bipolaron were obtained using the measured current between the gold and the Ag/AgCl electrodes that was recorded by chrono-amperometry simultaneously to the film absorbance. The voltage was applied in subsequent increasing steps as shown in Figure S2a. The current as a function of time was measured throughout the whole experiment and separated into the different voltage steps, as shown in Figure S2b. By integrating the current, we obtained the injected charge during each step, shown in Figure S2c. It can be noted that the current never decays completely to zero due to a residual current, and as a result, the injected charge increases linearly with time. To remove this contribution, we can fit a line and extrapolate it to time zero.[4] This gives us the amount of charge injected in the film during each subsequent voltage step. The total cumulative charge carrier density injected into the film as a function of voltage is shown in Figure S2d.

Having these values, we plot the absorbance at the spectral maximum for each species versus the injected charge carrier density, as shown in Figure S2e. To remove the spectral overlap between the species, the absorbance is found by multiplying the MCR concentration with the spectral component of each of the three species at all voltages. Considering that for lower doping levels (voltage applied > - 0.5 V) the number of bipolarons formed is negligible, it is reasonable to admit that in this range the injected carrier density is equal to the density of polarons, and equal to the depleted neutrals. With this assumption, we plotted the absorption at the maximum for the neutral and the polaron versus their density. The resulting plot was linearly fitted, and the slope obtained is equal to the absorption cross section of the polarons and neutrals. For the measurements in aqueous electrolyte, the maximum neutral absorption cross section is of $5.9 \cdot 10^{-16}\ cm^2$ while for the polaron it is $3.75 \cdot 10^{-16}\ cm^2$. Now the determination of the density of polarons for all voltages is straightforward, and hence, the density of bipolarons can be obtained from the remaining injected charge assuming that two injected charges are necessary to form one bipolaron. We thus repeated the procedure

[4] Y.Harima, T. Eguchi, K. Yamashita, Enhanncement of carrier mobilities in poly(3-methylthiophene(by an electrochemical doping, *Synth. Met.*, ***1998***, *95*, 69-74

and plotted the absorbance of the bipolarons versus their density, as shown in Figure S2f. Again, the linear fit of the curve was used to obtain the bipolaron absorption cross section, which values $5.27 \cdot 10^{-16}\ cm^2$. Finally, the MCR was re-run by fixing the spectral components to the absorption cross sections of the three species, which allowed to obtain their real density as a function of the applied voltage.

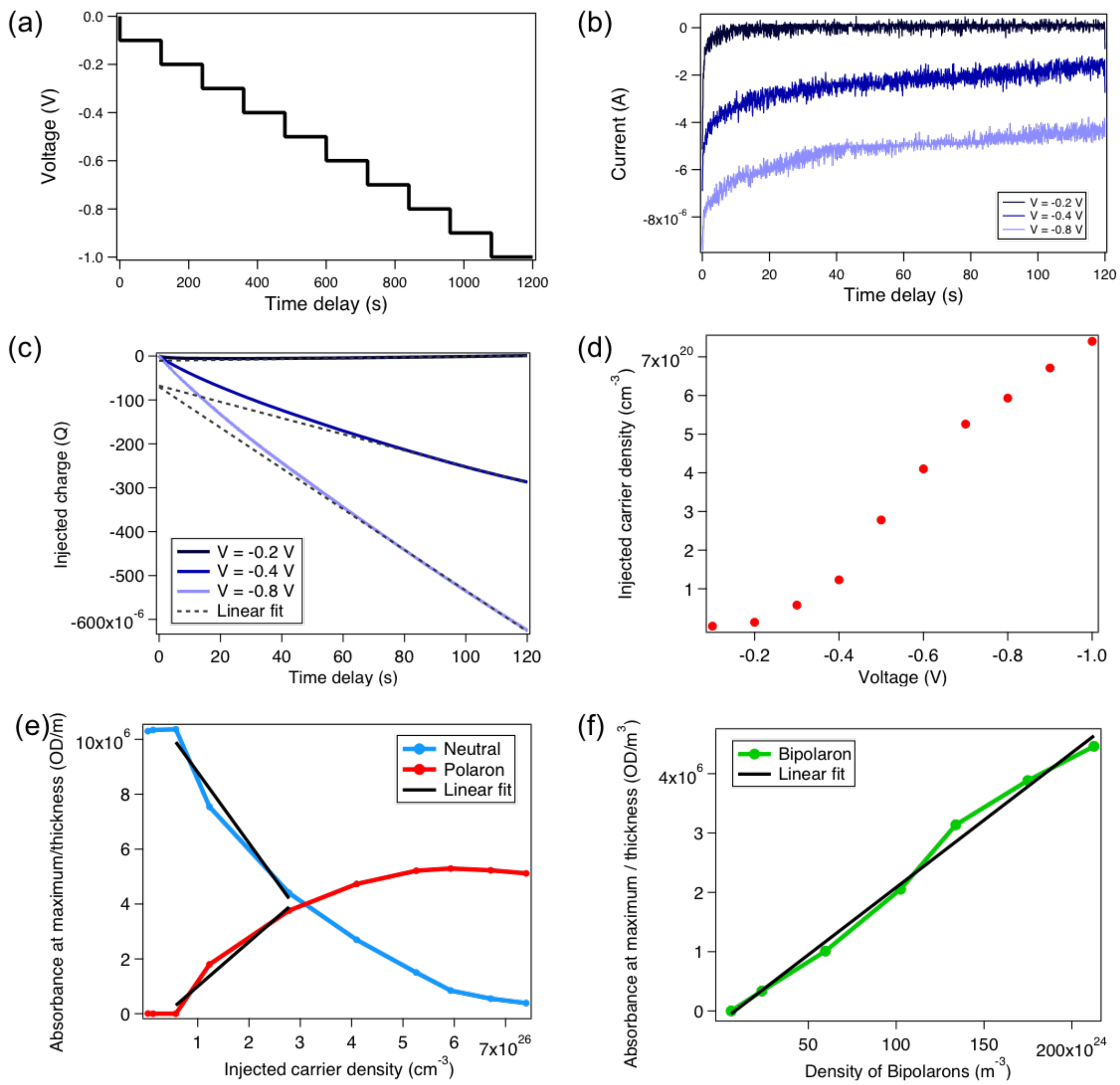

Figure S2 a) Voltage steps applied between the gold and the Ag/AgCl electrodes during the experiments. b) Current transients and the c) integrated injected charge at three selected voltages. d) Cumulative total injected carrier density calculated for all doping steps. e) Absorbance of the neutral and polaron bands at their maxima and f) absorbance of the bipolarons as a function of their density.

S6. Electrochemical doping of P3HT in $TBAPF_6$/acetonitrile electrolyte

To reach higher doping levels, the spectro-electrochemistry and THz measurements were repeated with $TBAPF_6$/acetonitrile electrolyte. The same procedure was used, but here the voltage range applied was between -0.5 V and -1.8 V. The same MCR analysis as in water electrolyte was performed on the spectro-electrochemistry data in order to extract the concentration of the neutral, polaron and bipolaron species. The absorption cross sections found were $4.9 \cdot 10^{-16}\ cm^2$ for the neutral, $3.2 \cdot 10^{-16}\ cm^2$ for the polaron, and $5.4 \cdot 10^{-16}\ cm^2$ for the bipolaron. With the exception of the neutral, the absorption cross section values are very similar to the ones measured in aqueous electrolyte. The discrepancy between the values found for the neutral is likely because the data was analyzed starting at a doping voltage of -0.5 V, where the neutral band is already less structured and represents amorphous P3HT domains that have a lower absorption cross section.

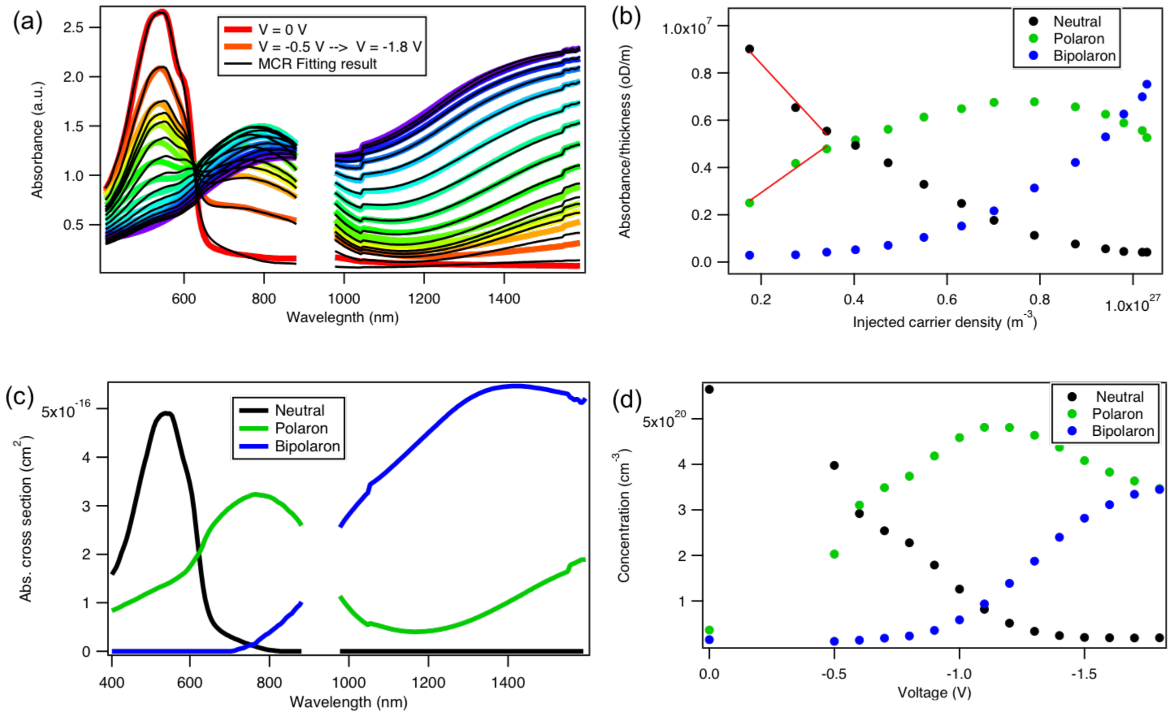

Figure S3 a) *In-situ* absorbance spectra of the spin-coated P3HT film at different applied voltages in 0.1M $TBAPF_6$/acetonitrile in steady state conditions. b) Absorbance of the neutral, polaron and bipolaron bands at their maxima as a function of injected charge density. c) Component spectra and d) voltage-dependent concentrations of the neutral, polaronic and bipolaronic species of electrochemically doped P3HT, as obtained from MCR analysis of the spectro-electrochemistry data.

S7. THz conductivity in electrochemically doped of P3HT in $TBAPF_6$/acetonitrile electrolyte

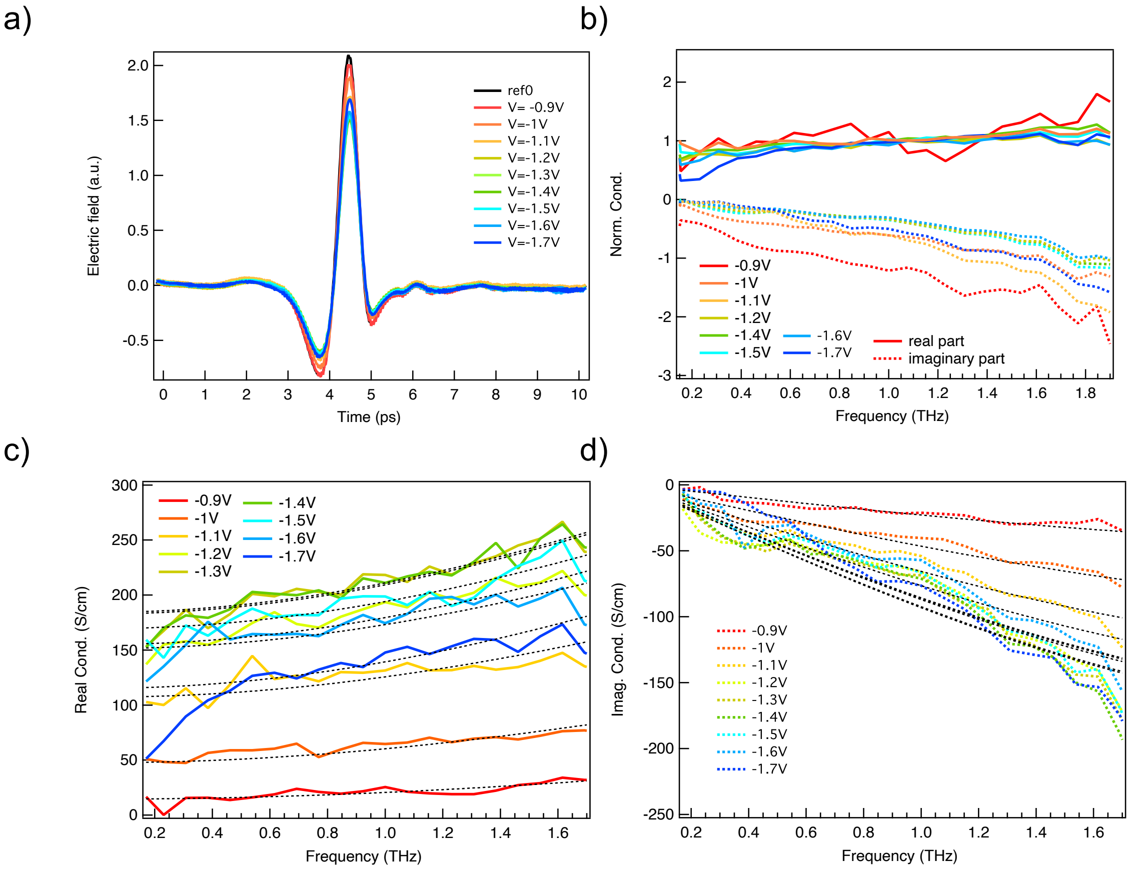

Figure S4 a) Transmitted time-domain THz waveform of electrochemically doped P3HT in 0.1M $TBAPF_6$/acetonitrile electrolyte at different oxidation potentials. b) Normalized real (solid) and scaled imaginary (dashed) parts of the complex conductivity at different applied voltages. c) Not normalized real and d) imaginary conductivity spectra. Black dotted lines show the Drude-Smith fits.

Table S1. Fitting parameters from the Drude-Smith model-based analysis of the complex THz conductivity spectra in electrochemically doped P3HT in acetonitrile electrolyte.

V versus Ag/AgCl (V)	ω_P (THz)	*N* ($x10^{19}$ cm^{-3})	τ (fs)	c_1
**-0.9**	350	6.7	20	-0.93
**-1**	530	15	20	-0.9
**-1.1**	656	23	20	-0.86
**-1.2**	761	31	20	-0.85
**-1.3**	809	35	20	-0.84
**-1.4**	809	35	20	-0.84
**-1.5**	774	32	20	-0.84
**-1.6**	737	29	20	-0.84
**-1.7**	737	29	20	-0.88
